# Does setup on rectal wall improve rectal cancer boost radiotherapy?

**DOI:** 10.1186/s13014-018-1011-4

**Published:** 2018-04-04

**Authors:** Jean-Paul J. E. Kleijnen, Bram van Asselen, Martijn Intven, Johannes P. M. Burbach, Marielle E. P. Philippens, Jan J. W. Lagendijk, Bas W. Raaymakers

**Affiliations:** Department of Radiotherapy, University Medical Center Utrecht, Heidelberglaan 100, 3584 CX Utrecht, The Netherlands

**Keywords:** Dose escalation, MRI, Mobility, Setup, Rectal wall, Position surrogate, Rectal cancer, Tumor, GTV, Boost

## Abstract

**Background:**

Rectal cancer patients that show a pathological complete response (pCR) after neo-adjuvant chemo-radiotherapy, have better prognosis. To increase pCR rates several studies escalate the tumor irradiation dose. However, due to lacking tumor contrast on online imaging techniques, no direct tumor setup can be performed and large boost margins are needed to ensure tumor coverage.

The purpose of this study was to evaluate the feasibility of performing a setup on rectal wall for rectal cancer boost radiotherapy, thereby using rectal wall nearby the tumor as tumor position surrogate.

**Methods:**

For sixteen patients, daily MRI’s were performed during 1 week of radiotherapy. On each of these images, tumor and rectum were delineated. Residual displacements were determined per surface voxel after setup on bony anatomy or nearby rectal wall and setup errors for both setups were compared. Furthermore for every rectal wall voxel nearby the tumor, displacement was compared with the closest tumor point and correlation was determined.

**Results:**

Mean (SD) setup error was 2.7 mm (3.3 mm) and 2.2 mm (3.2 mm) after setup on bony anatomy and rectal wall respectively. Nevertheless, similar PTV-margin estimates i.e. 95th percentile distances, were found; 8.0 mm. Also, a merely moderate correlation; ρ = 0.66 was found between rectal wall and tumor displacement. Further investigation into tumor and rectal mobility differences showed that the rectal wall lacks appropriate anatomical landmarks to find true displacements, especially to capture motion along the rectal wall.

**Conclusions:**

Setup on rectal wall slightly reduces mean setup errors but requires a similar PTV-margin as compared to setup on bony anatomy. Rectal mobility might be similar to tumor mobility, but due the absence of anatomical landmarks in the rectum, displacements along the rectal wall are not detected on current online imaging. Therefore, to further reduce tumor position uncertainties, direct or indirect online tumor visualization is needed.

## Background

The treatment paradigm for rectal cancer is changing with the introduction of organ sparing alternatives for patients responding well to neoadjuvant therapy [1]. As the response to neoadjuvant radiotherapy is dependent on the irradiation dose, several dose escalation studies using external beam radiotherapy or brachytherapy are currently being conducted [2, 3].

**Fig. 1:**
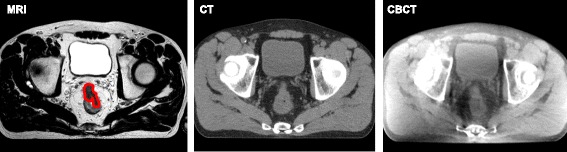
Transverse images of the lower abdomen of a rectal cancer patient using different imaging modalities. The images are of the same patient, depicting the same location through the rectal tumor. On the left; MRI, in the middle; computed tomography (CT) and at the right; CBCT. The tumor is delineated in red on the MR image. Note: on CT and CBCT images, there is contrast between the rectum and surrounding mesorectal fat, but no contrast between the rectum and the tumor

External beam radiotherapy dose escalation is challenging as the target, the rectal tumor, is not visible on online cone beam computed tomography (CBCT) imaging (Fig. 1). One way to overcome this problem is to perform a setup on a surrogate for the rectal tumor position on the CBCT. Traditionally, this is done by using the pelvic bony anatomy as a position surrogate for setup [4]. Since rectal tumors originate from the rectal wall, which has visibility on CBCT, the rectal wall might be a better position surrogate for the tumor due to it’s close proximity. Due to this proximity, similar mobility for tumor and rectal wall is often assumed in literature [5–7], but was never investigated.

To investigate the feasibility of a setup on rectal wall, magnetic resonance imaging (MRI) can be used, since it provides contrast for both tumor and rectal wall (Fig. 1). Mobility of these structures has to be investigated separately since the contrast of the tumor, which is not present on the online CBCT imaging, might influence the derived mobility of the rectal wall. When rectal wall and tumor mobility are investigated separately, rectal wall mobility on CBCT and MRI imaging will be similar.

As with every surrogate, some uncertainty in tumor position will remain, whether this is a standard setup on bony anatomy or a setup on rectal wall. To quantify the benefit of using the nearby rectal wall as position surrogate, errors in tumor position after both a setup on rectal wall and a setup on bony anatomy can be compared.

The purpose of this study was to test the feasibility of using the nearby rectal wall as position surrogate for the rectal tumor during rectal cancer boost radiotherapy. We therefore quantified how large setup errors were in case of a set-up on nearby rectal wall as compared to a traditional set-up on bony anatomy, by using consecutive MRI scans. Additionally, we determined the correlation between mobility of the tumor and the nearby rectal wall.

## Methods

### Data acquisition

Sixteen patients, diagnosed with a local rectal tumor (T2-3, N0-1, M0-1) were included after written informed consent was obtained. Patients received five daily radiotherapy fractions of 5 Gy to the clinical target volume (CTV), i.e. the mesorectum and the iliac, obturator and pre-sacral lymph nodal regions. Within a medical research ethics committee approved study, these patients were scanned daily on a 1.5 T MRI scanner (Philips Intera, Philips medical systems, Best, The Netherlands), prior to each fraction of radiotherapy resulting in five MRI scans per patient. For 4 patients, one MRI scan could not be made due to logistical reasons, resulting in a total of 76 MRI scans to be analyzed. Patients were scanned in treatment position (supine) on a flat table top using a 4 element body coil. Scanning protocol consisted of T2 weighted (T2w) and diffusion weighted imaging (DWI). T2w imaging was acquired on a resolution of 0.62 × 0.62 mm^2^, slice thickness of 4.0 mm, and the number of slices was 30. A repetition time (TR) of 3713 ms and an echo time (TE) of 120 ms were used. The scan time was 6:21 min. DWI was performed with a resolution of 1.87 × 1.87 mm^2^ and a slice thickness of 4.0 mm. The number of slices was 40 and b-values of 0, 200 and 800 [s/mm^2^] were chosen. The scan time was 4:37 min.

To evaluate the mobility of the nearby rectal wall and the tumor separately, the rectum and the tumor were delineated on each MRI scan by an experienced radiation oncologist. Delineations were made on the T2w scan with DWI next to it as a guideline, using our in-home developed multi-modality delineation software [8].

### Setup on bony anatomy

For each patient, the first scan was considered as the reference scan and rigidly registered to each daily scan, mimicking the set-up on bony anatomy which is performed prior to each fraction of radiotherapy. These rigid registrations were done using the ‘Euler’ registration of the *Elastix* toolkit [9]*.* Registration accuracy was checked by visual inspection of bony alignment and errors were found to be limited to one voxel.

Using the transformation matrix of the registration, delineations of the tumor and rectum of the reference scan were propagated to each daily scan. To determine the non-rigid displacements, distances between the reference delineation and all daily delineations were determined per surface voxel, for rectum and tumor separately. These distances were determined using the bidirectional local distance measure as described by Kim et al. [10].

By mapping the repeated delineations, we were able to evaluate rectal mobility independently of tumor mobility. In this way we can use MRI, where both rectal and tumor contrast is present, to evaluate a CBCT guided situation, in which merely rectal wall contrast is present. To determine the errors after setup on bony anatomy, mean and standard deviation were determined of all tumor displacements. Furthermore a cumulative distribution function of these displacements was calculated. The 95th percentile of this cumulative distribution function was taken as a planning target volume (PTV) margin estimate for compensating these bony anatomy setup errors.

### Setup on nearby rectal wall

**Fig. 2:**
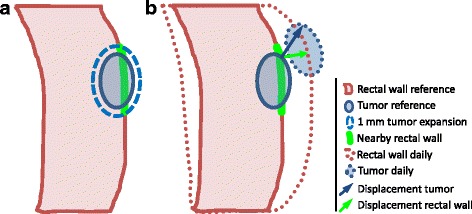
Schematic sagittal overview of used method. **a** The nearby rectal wall (green) was defined as that part of the rectal wall (red) that lies within a 1 mm expansion (dashed blue) around the tumor (blue). **b** For both tumor and nearby rectal wall, distances between the reference (solid lines) and daily (dotted lines) delineations were determined. For all points of the nearby rectal wall the corresponding i.e. nearest, tumor point was determined. As an example, displacements for a pair of corresponding points of the position surrogate and tumor are shown by the arrows. As measure of how well position surrogate and tumor mobility are in agreement, Pearson’s correlation was determined between displacements of each pair of corresponding points, i.e. the correlation between the green and blue arrow, for all points of the nearby rectal wall

To determine the errors after a setup on nearby rectal wall, also such a setup was performed. We therefore defined a position surrogate based on the rectum delineation on the reference scan, taking into account only that part of the rectal wall close to the tumor. More precisely, by taking that part of the rectal wall that lies within a 1 mm expansion of the tumor. A graphical example of how this position surrogate was defined can be seen in Fig. 2a.

The rectal wall of the reference scan is aligned with the rectal wall on each daily scan and the reference tumor delineation was transformed accordingly. This alignment was done for each day, by applying the mean displacement of the position surrogate, i.e. the rectal wall nearby the tumor, as a setup correction. This results in the optimal rigid alignment of the reference and daily rectal wall nearby the tumor, whereby the mean distance between the reference and daily rectal wall after alignment becomes zero.

After this setup on nearby rectal wall, tumor displacements were determined similar to the setup on bony anatomy i.e. the local displacements between the reference delineation and all daily delineations were determined per surface voxel using the bidirectional local distance measure. We then determined the errors after setup on nearby rectal wall by determining the mean and standard deviation of all displacements, similar to the setup on bony anatomy. Furthermore, a cumulative distribution function was calculated to determine a PTV-margin estimate for compensating the setup errors for a setup on nearby rectal wall.

To analyze the performance of the setup methods, both setup errors and PTV-margin were then compared between setup on bony anatomy and nearby rectal wall.

### Comparison of tumor and nearby rectal wall mobility

To define how well tumor and nearby rectal wall mobility are in agreement, correlation between displacements of tumor and nearby rectal wall was determined. Therefore the residual displacements after bony set-up were compared. This was done by a voxel-by-voxel comparison of the displacements of both tumor and nearby rectal wall. Hereby, the displacement of each voxel of nearby rectal wall was compared to the displacement of the closest voxel of the tumor reference delineation as can be seen in Fig. 2b. The tumor and nearby rectal wall displacements between the reference delineation and all daily delineations were compared.

### Statistical analysis

To determine the effectiveness of a setup on nearby rectal wall we performed the following statistical analysis.

To check for differences in setup errors for the tumor between a setup on bony anatomy and a setup on nearby rectal wall, we performed a two tailed paired t-test. Furthermore the Pearson’s correlation coefficients between tumor and position surrogate displacements were determined. All *P*-values; *p* ≤ 0.05 were considered as significant.

## Results

**Fig. 3:**
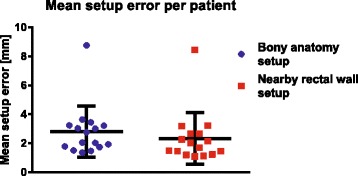
Scatter plot of the mean setup error per patient for setup on bony anatomy and nearby rectal wall. The median is indicated with the black horizontal line and the standard deviation by the vertical whiskers

We determined the residual tumor displacement after setup, i.e. the mean setup error, for setup on bony anatomy and nearby rectal wall. The mean setup errors were 2.7 mm (standard deviation (SD) 3.3 mm) and 2.2 mm (SD 3.2 mm) for setup on bony anatomy and nearby rectal wall, respectively. Mean setup errors per patient can be seen in Fig. 3.

**Fig. 4:**
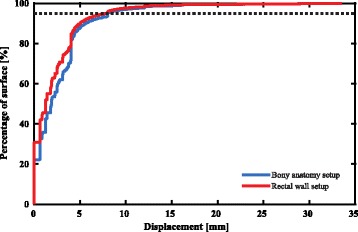
Cumulative distribution plot of residual displacements i.e. setup errors, after setup on bony anatomy and nearby rectal wall. The level of the 95th percentile of the setup error is indicated by the dashed line

In order to assess the required margins to compensate for setup errors after setup on bony anatomy or nearby rectal wall, cumulative distribution functions were determined (Fig. 4). The required PTV-margin estimate, i.e. the 95th percentile of the residual displacements, was 8.0 mm for both the setup on bony anatomy and nearby rectal wall.

**Fig. 5:**
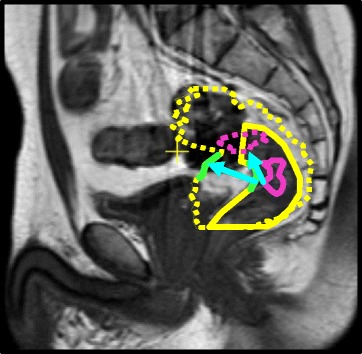
Delineations of the rectal wall (yellow), nearby rectal wall (green) and tumor (purple) on a sagittal MRI scan. The dashed delineations are overlaid from a different treatment day. Note the difference in amount of displacement and direction, between tumor and nearby rectal wall (cyan arrows)

For the comparison of displacement of tumor and nearby rectal wall, a moderate correlation between the two was found; *r* = 0.66 and *p* ≤ 0.001. An example of mismatch between tumor and nearby rectal wall displacement can be seen in Fig. 5. Rectal filling has changed between the two scans. When evaluating the displacement, the rectum seems to extend in the transverse direction. However the tumor position also reveals displacement in cranial direction. Therefore points on tumor and nearby rectal wall that were initially close to each other, show very different displacements when evaluated separately, as indicated by the arrows in the figure.

## Discussion

This is the first study to investigate the feasibility of performing a setup on nearby rectal wall for boost delivery, thereby using the rectal wall close to the tumor as a position surrogate for tumor position. We furthermore investigated the correlation between daily tumor and nearby rectal wall displacements. For a tumor position surrogate to be feasible, high correlations are needed to provide accurate tumor positions. The lower the correlations, the larger and more frequent errors in tumor position will be.

Although a moderate correlation between nearby rectal wall and tumor position was found, only a slight reduction in setup errors was observed; the mean setup error was reduced by 0.5 mm (from 2.7 to 2.2 mm). The 95th percentile distance, which is the PTV-margin estimate that includes 95% of the target surface, was similar for both setup methods. This suggests that no reduction of PTV-margin can be achieved by performing a setup on nearby rectal wall over a setup on bony anatomy. Thus, despite the slightly smaller average setup error, similar margins are still required.

**Fig. 6:**
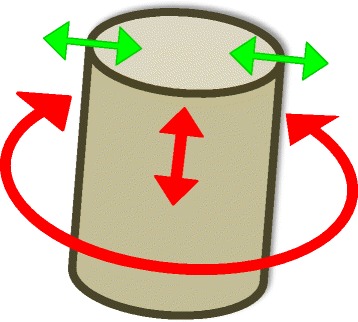
Schematic explanation of undetected mobility in absence of imaging contrast. The rectum is simplified as a tube, having only contrast with its surroundings and no internal anatomical landmarks, similar to the contrast of the rectal wall on CBCT. Distensions can be detected in this situation since displacements occur perpendicular to the image contrast (green arrows). However, rotations as well as displacements along the tube, e.g. cranial-caudal movement in case of the rectum, cannot be observed detected since they occur parallel to the image contrast (red arrows)

The merely slight reduction of setup errors and the intermediate correlation coefficient found indicate that either tumor and rectal wall move more or less independently or that the observed rectal wall displacement on imaging is an over- or under-estimation of true rectal wall displacement. Further investigation of individual cases shows the latter is the case, as can be seen in figure 5. Displacement was defined as the distance between repeated delineations. In the example in Fig. 5, we observe tumor displacement in cranial-caudal (CC) direction, resulting in displacement along the rectal wall contrast border. When displacement for the rectum is considered, this displacement in cranial-caudal direction will not be captured, since no contrast for the rectal wall is available in the direction along the rectal wall. Nor are there anatomical landmarks present, except for the tumor itself, that will guide image registration in an online setting. This results in a difference in observed tumor and nearby rectal wall displacement. A more general and schematic explanation can be seen in Fig. 6. When the rectum is simplified as a tube, in absence of landmarks on imaging as for instance on CBCT, distensions can be seen on imaging because they occur perpendicular to the image contrast. However both rotations and displacements along the rectum cannot observed since they occur parallel to the image contrast.

Determining the rectal wall displacement by matching delineations of the rectum or registering online CBCT images to prior imaging, will therefore result in an unrealistic solution in such a situation. In this paper, we have chosen to determine displacements by matching delineations using a local bi-directional distance measure, mimicking a CBCT guided situation, to evaluate whether the observed nearby rectal wall displacements are in concordance with tumor displacements. When we would apply image registration to determine displacement of the rectal wall between the repeated MRIs, this displacement would be estimated (more) correctly since the tumor in situ acts as an anatomical landmark for the registration. However, since in current clinical practice CBCT neither provides this tumor contrast nor other anatomical landmarks, an image registration based method cannot be used to investigate the feasibility of a setup on nearby rectal wall for CBCT guided (adaptive) radiotherapy due to the presence of tumor contrast in our MRI scans.

Several studies were done to derive (estimates of) tumor PTV-margins based on rectal motion statistics, thereby making the assumption that tumor and rectal wall moves alike [5–7]. All these studies were performed on CBCT, which does not provide adequate contrast to assess true rectal wall displacement. When rectal motion statistics are characterized in such a setting, correlation between rectal and tumor motion is limited as shown by this study.

A study that both quantified tumor and rectal mobility by Brierley et al. reported the largest tumor motion in CC direction, which might explain the poor performance of a rectal wall-based tumor position surrogate [11]. Unfortunately, the study by Brierley could not quantify mobility in CC direction for the rectum due to limitations of their methodology, and therefore could not detect potential differences in tumor and observed rectal mobility.

Although the nearby rectal wall might have the same mobility as the tumor itself, this motion cannot be found due to the lack of anatomical landmarks along the cylinder shaped rectum, especially in CC direction. Since tumor mobility is highest in CC direction, it is not advised to define tumor PTV-margins based on rectal wall mobility. Alternatively, tumor positions can be found online by direct tumor visualization using online MRI as proposed by Lagendijk et al. or by indirect tumor visualization, i.e. by inserting fiducial markers into the tumor as described by Moningi et al. [12, 13].

## Conclusions

A setup on nearby rectal wall reduces setup errors slightly on average, but requires a similar PTV-margin as compare to a setup on bony anatomy. Although the mobility of the nearby rectal wall might be similar to the tumor mobility itself, one cannot use rectal wall mobility as surrogate for tumor mobility. This is explained by the fact that displacement of the rectal wall and tumor along the direction of the rectal wall will not be detected due to the absence of anatomical landmarks. Observed rectal wall displacement on imaging can therefore be an over- or underestimation of tumor displacement. To further reduce uncertainties in rectal cancer boost radiotherapy, direct or indirect online tumor visualization is needed.

## Abbreviations

CBCT: Cone-beam computed tomography
CC: Cranial-caudal
CT: Computed tomography
CTV: Clinical target volume
DWI: Diffusion weighted imaging
MRI: Magnetic resonance imaging
pCR: Pathological complete response
PTV: Planning target volume
SD: Standard deviation
T2w: T2 weighted
TE: Echo Time
TR: Repetition time
